# MEK inhibitors - novel targeted therapies of neurofibromatosis associated benign and malignant lesions

**DOI:** 10.1186/s40364-021-00281-0

**Published:** 2021-04-16

**Authors:** Anja Harder

**Affiliations:** 1 Institute of Pathology, Martin Luther University Halle-Wittenberg, Magdeburger Str. 14, 06120 Halle (Saale), Germany; 2 Institute of Neuropathology, University Hospital Münster, Münster, Germany; 3 Faculty of Health Sciences, Joint Faculty of the Brandenburg University of Technology Cottbus – Senftenberg, the Brandenburg Medical School Theodor Fontane and the University of Potsdam, Potsdam, Germany

**Keywords:** MEK inhibitor, Neurofibromatosis, NF1, NF2, Schwannomatosis, LGG, Neurofibroma, MPNST, Glioblastoma, RASopathy

## Abstract

MAP/ERK kinase 1 and 2 (MEK 1/2) inhibitors (MEKi) are investigated in several trials to treat lesions that arise from pathogenic variants of the *Neurofibromatosis type 1 and type 2* genes (*NF1*, *NF2*). These trials showed that MEKi are capable to shrink volume of low grade gliomas and plexiform neurofibromas in NF1. Targeting other lesions being associated with a high morbidity in NF1 seems to be promising. Due to involvement of multiple pathways in NF2 associated lesions as well as in malignant tumors, MEKi are also used in combination therapies. This review outlines the current state of MEKi application in neurofibromatosis and associated benign and malignant lesions.

## Background

A targeted therapy of Neurofibromatosis (NF) ideally would start early to inhibit tumor development and, at best, would cure the disease. Soon restoration of the mutational effect would raise the amount of functional protein and compensate impaired functions. Currently, substantial improvement has been made regarding targeted therapies by using MAP/ERK kinase 1 and 2 (MEK 1/2) inhibitors (MEKi) to block RAS-MAPK overactivation and to minimize the mutational effect on the somatic level. Here, the published status of MEKi therapies in NF with special respect to Neurofibromatosis type 1 associated lesions is reviewed.

### RAS-MAPK signaling cascade and selective MEK1/2 inhibition

The mitogen-activated protein kinase kinase kinase (MAP 4 K) hierarchical pathway (RAS-RAF-MEK-ERK) is important for proliferation, differentiation and survival of cells and is overactive in many cancers [1]. Cell surface tyrosine kinase receptors, Ca2+, protein kinase C or G protein-coupled receptor activate nucleotide guanosine triphosphate (GTP)ase bound Kirsten rat sarcoma virus (RAS/MAP 4 K) which transduces the extracellular signal to many profound substrates through phosphorylation of the following intracellular kinases: rapidly accelerated fibrosarcoma (RAF/MAP 3 K or MAPKK), MAP/ERK kinase 1 and 2 (MEK 1/2 / MAP 2 K), and extracellular signal-related kinase (ERK/MAPK). Activated ERK finally transfers the signals into the cellular transcription network [1]. Phosphorylation of protein kinases and substrates is a highly significant regulatory mechanism in cells. Therefore, inhibition of phosphorylation for therapy of disease can be expected to lead to multiple side effects.

Enzymes MEK1 and MEK2 are conserved, important dual specificity serine/tyrosine protein kinases of 44 and 45 kDa molecular weight. They can be specifically targeted by inhibitors (MEKi) which arrest MEK1/2-dependent signaling in a highly selective way. MEK proteins of the MEK family structurally share an amino-terminal domain, a conserved kinase domain, and a carboxyl-terminal domain [2]. MEK1/2 are encoded by *MAP 2 K1* (15q22.31) and *MAP 2 K2* (19p13.3). Many inhibitors block components of the RAS-MAPK signaling cascade but MEKi inhibitors were the first selective ones that effectively approached patients. Thus, trametinib was the first clinically successful MEKi used for melanoma with *BRAF* pathogenic variants [3]. Targetable catalytic processes occur within ATP binding site of the kinase domain. MEKi act non-competitive or competitive with ATP, but only those that bind allosteric to the ATP binding site are very specific [2].

Currently, MEKi therapy is limited by two major problems: drug resistance and toxicity.

**Fig. 1:**
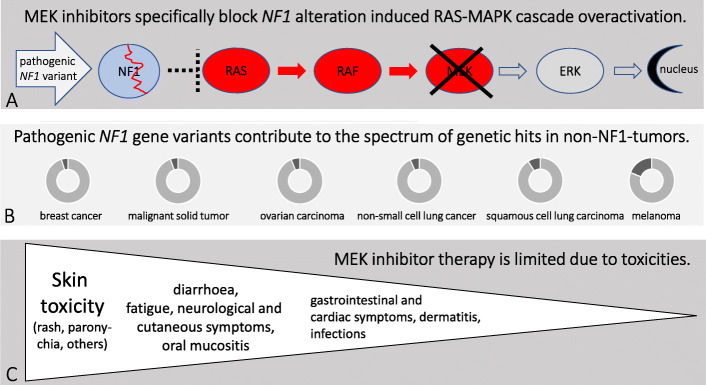
Principles of MEK inhibition in NF1 associated lesions. Legend: **a** *NF1* pathogenic variants induce overactivation of the MAPK signaling cascade. Specific MEK inhibition blocks phosphorylation of ERK and subsequent signal transduction to the transcription network of the nucleus. **b** Apart from Neurofibromatosis type 1, somatic pathogenic *NF1* gene variants occur in non-NF1 associated tumors and can be targeted by MEK inhibitors. **c** MEK inhibition is associated with side effects which occur at different percentages

Resistance due to reactivation of RAS-MAPK signaling can arise from alterations of *RAS*, *RAF*, *NF1*, or *MEK*, from reactivation of upstream receptor tyrosine kinases (e. g. hepatocyte growth factor (HGF) / HGF receptor (MET) signaling) due to adaptation, from activated parallel pathways (PI3K, STAT, Hippo and signal transducer and activator of transcription (STAT) signaling, loss of *PTEN or PP2A*) as well as from activated transcription factors to control phenotypes and metabolism [4, 5]. Thus, pathogenic variants in *MAP 2 K1* or *MAP 2 K2* influence sensitivity to MEKi [6]. Hence, acquired pathogenic variants such as *MEK1*^*V211D*^ can induce resistance to allosteric MEKi, which may be overcome by a new class of adenosine triphosphate (ATP)-competitive MEKi [7]. In principle, allosteric ATP-non-competent compounds bind MEK adjacent to the conserved ATP binding pocket. Non-competitive binding induces enzyme inactivation due to a change of protein conformation: the activation loop that needs to be phosphorylated at serine residues S127 and S221 to enable a complete biological activity, remains incompletely phosphorylated. MEKi trametinib also blocks S218. Subsequently, the catalytically inactive state of protein kinase MEK is locked and activated RAF kinases are not able to pass activity. An overactive (e.g. *BRAF* mutated) RAF kinase that is not able to phosphorylate MEK leads to suppression of downstream signaling (via phosphorylation of ERK) and blocks pathway overactivation. Similarly, upstream overactivated (e. g. *RAS* or *NF1* mutated) RAS cannot transduce oncogenic signals when MEK is blocked (Fig. 1a). However, upstream kinases can stimulate other effectors and induce drug escape: For example, KRAS can stimulate the phosphatidylinositol 3-kinase (PI3K). Therefore, in *KRAS* mutant cancers additional *PI3K* mutations lead to reduced sensitivity to MEKi. Those cancers require combination therapy, and identification of biomarkers is important [8]. Trametinib is one of those MEKi that is currently tested in combination with others inhibitors [9]. During therapy drug resistance can also occur due to negative feedback signaling downstream of RAS [4]. To overcome those adaptive mechanism novel drugs such as trametiglue that enhance binding are investigated [9].

Inhibitors of MEK1/2 are currently used for therapy of *BRAF* mutated as well as *Neurofibromatosis type 1 (NF1)* mutated, *KRAS* and *NRAS* mutated tumors including treatment of RASopathies (Fig. 1). Of those, trametinib, cobimetinib, selumetinib, binemetinib and mirdametinib are in use to treat patients. As reported by the database of the National Cancer Institute (“ClinicalTrials.gov”) four MEKi studies have been completed for neurofibromatoses: NCT02096471, NCT02124772, NCT01885195, and NCT03649165. They involved PD-0325901 (mirdametinib), trametinib, dabrafenib, and MEK162 (binimetinb) in phase 1 and 2 studies and included individuals with NF1-associated plexiform neurofibromas and other cancers harboring V600 mutations or RAS/RAF/MEK activated tumors. Inhibition of MEK1/2 by selumetinib affects MAP 2 K dependent pathways in *NF1* mutated inoperable plexiform tumors and was recently approved by the U. S. Food and Drug Administration (FDA). Other MEKi therapies target patients with ganglioglioma, non-small cell lung carcinoma, pilocytic astrocytoma, pleomorphic xanthoastrocytoma, malignant solid tumor, hematologic disorders, and colorectal cancer [10].

### RAS-MAPK signaling cascade in Neurofibromatosis

Neurofibromatosis (NF) type 1 (NF1), type 2 (NF2) and type 3 (Schwannomatosis) are inherited neurocutaneous tumor syndromes that affect multiple organs and share development of multiple benign peripheral nerve sheath tumors eponymous for the disease. They are caused by germline pathogenic loss-of-function variants of tumor suppressor genes on 17q11.2 (*Neurofibromatosis type 1 gene, NF1)*, on 22q11.2 (*Neurofibromatosis type 2 gene, NF2)*, and on 22q.23 (*SWI/SNF related, matrix associated, actin dependent regulator of chromatin, subfamily b, member 1 gene, SMARCB1* and on 22q11*.21 (Leucine zipper like transcription regulator gene, LZTR1*). Besides germline events in these syndromes, somatic pathogenic variants of the involved genes can also arise in several sporadic, non-NF associated cancers (Fig. 1b) [11, 12]. NF belong to RASopathies. RASopathy-associated tumors are treated in trials using MEKi such as cobimetinib (NCT02639546). In most RASopathies germline pathogenic variants in genes encoding RAS pathway proteins affect functions upstream of MEK1/2. Thus, drug escape can be anticipated by upstream genetic alterations [13]. A new NCI initiative (“Advancing RAS/RASopathy (ART)”) aims to develop therapeutic strategies for RASopathy associated lesions [14].

In NF2, therapy of brain tumors precedes therapy of peripheral tumors since associated vestibular schwannomas, ependymomas and meningiomas lead to more severe complications. Therefore, endpoints of trials for NF2 associated tumors differ compared to other cancer studies [15]. *NF2* pathogenic germline variants of moesin-ezrin-radixin-like protein called merlin or schwannomin effect not only RAS-MAPK signaling but also tyrosine receptor kinases and many other downstream pathways underlining a complex multi-suppressor function of merlin [2, 16]. Nevertheless, pre-clinical studies extensively studied MEKi. A large study analyzed MEKi selumetinib, trametinib, PD0325901, MEK162, cobimetinib and refametinib in NF2-associated merlin-deficient schwannoma cells and mouse models and identified trametinib, PD0325901 and cobimetinib to be the most effective as well as uncovered resistance mechanisms [3]. In a *NF2*-mutation associated tumor model application of MEKi trametinib alone as well as in combination with vistusertib was effective [6]. Somatic pathogenic variants can occur additionally to *NF2* gene variants such as in *AKT1* (e.g. *AKT1*^*E17K*^ variant) which highlights the importance of tumor genome analysis prior to a targeted therapy [7]. Consequently, merlin deficiency can be rescued not only by MEKi but several other drugs and multiple alternative ways give rise to drug resistance in patients. Therapeutics that targeted single tyrosine kinases in trials were not successful so far [15, 17]. NF2-associated tumors, although benign, need a specific multi-target approach explaining why FDA-approved systemic MEKi therapies are not established and why (radio) surgery in NF2 is still a successful first line approach [18]. Currently, an open trial (SEL-TH-1601, NCT03095248) investigates response rate of NF2-associated tumors by selumitinib. Ongoing trials investigate MEKi selumitinib and cobimetinib for NF2-tumor-associated hearing loss and MEKi trametinib in combination therapy for aggressive and recurrent meningiomas.

Currently, no trial exists that investigates any kind of therapy of Schwannomatosis. Associated schwannomas show a combination of “first and second hits” of *SMARCB1*, *LZTR1*, *NF2* and others. A complex inactivation of different tumor suppressor genes leads to involvement of several pathways in development of benign tumors. As in NF2, more than RAS-MAPK signaling would need to be targeted.

In contrast, the RAS-MAPK signaling cascade seems to be a very promising target in NF1, at least in benign nerve sheath tumors, low grade gliomas and non-tumor lesions since RAS-MAPK activation is the main pathomechanism. As there is rapidly growing knowledge, MEKi therapy in NF1 is reviewed in the following sections.

### RAS-MAPK signalling cascade in Neurofibromatosis type 1 (NF1)

**Fig. 2:**
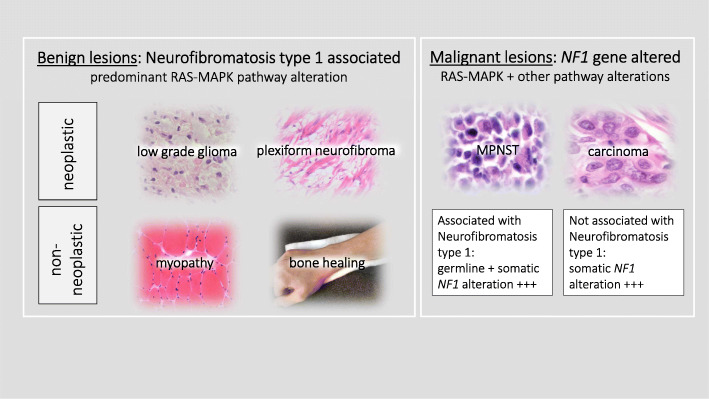
MEK inhibitors neutralize pathogenic *NF1* mutations. Legend: Main applications MEK inhibitors: MEKi can be principally applied for NF1 associated benign lesions or malignant tumors that harbor *NF1* gene pathogenic variants

Among neurofibromatoses, NF1 is most frequent and results from germline pathogenic variants of the *NF1* gene in about 50% of cases. NF1 is associated with a broad spectrum of symptoms including benign peripheral nerve sheath tumours (neurofibromas), café-au-lait macules (CALM), skinfold freckling, iris Lisch nodules, low grade gliomas, bone malformation and others [19, 20]. Clinical diagnosis of NF1 is defined by NIH criteria that are revised in 2021 to support differentiation from related syndromes [21]. NF1 patients are at increased risk for malignant transformation of neurofibromas. Benign lesions such as CALM, pseudarthrosis, and benign tumors arise from a “second (somatic) *NF1* hit” followed by loss of function of the gene product neurofibromin. The central, highly conserved guanosine triphosphatase (GTPase) activating protein (GAP)- related domain (GRD) of neurofibromin is crucial to downregulate RAS in many cells. *NF1* pathogenic variants that involve important binding sites of GRD dramatically reduce GAP activity of RAS [22, 23]. Essential regulation of RAS via GRD has prompted therapeutic targeting of the RAS-MAPK signaling cascade in NF1 long ago. In malignant, sporadic non-NF1-associated tumors such as pheochromocytoma, lung adenocarcinoma, breast cancer, ovarian cancer, glioblastoma and many others, somatic pathogenic variants of *NF1* are also important targets. MEKi have been investigated in several human and animal NF1 studies and to date trametinib and selumitinib are used in nearly 20 ongoing studies in NF1 patients. Although to date binimetinib, cobimetinib, trametinib, selumitinib, and mirdametinib (PD0325901) are investigated in trials, only selumitinib is FDA approved for plexiform neurofibromas. To summarize, MEKi can be used to neutralize a pathogenic *NF1* gene variant in following modes (Fig. 1a, b; Fig. 2):

- In NF1 patients, MEKi are effective in blocking RAS-MAPK overactivation in benign tumors (low grade gliomas, neurofibromas) that display a “first” and a “second (somatic) *NF1* hit” (Knudson’s hypothesis).
- In NF1 patients, MEKi might also be very useful to block RAS-MAPK overactivation in non-tumor lesions. Promising pre-clinical approaches demonstrated positive effects on bone lesions and fracture healing (see below). Even NF1 associated myopathic features or intimal proliferation (neointima) are successfully targeted or rescued with MEKi such as PD0325901 in murine models [24, 25].
- In tumors being unassociated with NF1, pathogenic variants of the *NF1* gene occur additionally to other genetic events on the somatic level. Here, MEKi need to be applied within combination therapy. Even in NF1-associated malignant tumors such as in MPNST, multi-step mutational processes afford combination therapy.

MEKi therapy remodels the kinome activity and gene expression in *NF1* mutant tumor cells [21]. Therefore, it is not surprising that MEKi therapy leads to multiple toxicities among which skin toxicities are most common and require dose adjustments (Fig. 1c). Detailed side effects have been published and recommendations for management have been compiled by the Clinical Care Advisory board of the Children’s tumor Foundation [26]. However, long term experience onof continuous therapies is missing.

### MEKi treatment of neurofibromas in Neurofibromatosis type 1

NF1 associated childhood plexiform neurofibromas occur in up to 50% of NF1 patients. They are congenital, can undergo malignant change and cause severe complications by invading neighboring structures. Inhibition of NF1 associated nerve sheath tumor growth by MEKi PD0325901 has been shown in extensive human and murine, in-vitro and pre-clinical studies [27]. For inoperable plexiform neurofibromas, MEKi treatment is now a valuable option, and response is standardized by volumetric MRI measurements [28]. Meanwhile several trials and single case studies using selumitinib, trametinib or PD-0325901 reported size reduction of plexiform and/or spinal neurofibromas in children with NF1 [29–37]. For instance, following up promising data from a phase 1 study, Gross and co-workers recently described durable tumor shrinkage in NF1 patients with plexiform neurofibromas with selumitinib (NCT01362803). They reported partial response in 70% of cases, durable responses over ≥1 year in 28 cases, clinical benefits, but also disease progression in 6 cases [36]. Selumetinib was also successfully applied in NF1 patients with spinal neurofibromas and was shown to reduce tumor burden, effect on the spinal canal, cerebrospinal fluid distribution, and spinal cord shape in 18 of 24 patients [38]. In one pilot study selumetinib is applied to reduce the size and number of neurofibromas in adult NF1 patients (NCT012839720). In summary, MEK inhibitors effectively decrease volume of plexiform neurofibromas and may be also be beneficial in cases with a high cutaneous neurofibroma burden or complicated spinal neurofibromas.

### MEKi treatment of low grade glioma in Neurofibromatosis type 1

MEKi selumetinib and trametinib are currently employed in trials for low grade gliomas (LGG) including NF1 patients (NCT03363217, PNOC021, NCT03871257, NCT04166409, NCT04576117, NCT033263388, NCT01089101). LGG are common brain tumors in children. About 20% of NF1 patients develop brain tumors of which pilocytic astrocytoma of the optic pathway is the most common [39]. Only 2–3% of NF1 patients with optic gliomas need standard chemotherapy [21]. Clinical trials with MEKi are implemented only for recurrent or refractive disease. Selective MEKi are clearly superior to multikinase inhibitors [40]. A multi-center study of 18 LGG cases including 8 NF1-related tumors demonstrated disease control in all patients with progressive *BRAF*, fibroblast growth factor receptor 1 (*FGFR1)* or *NF1* mutated tumors using trametinib [41]. NF1-associated tumors comprised posterior fossa and midline pilocytic astrocytoma as well as other tumors which showed typical DNA methylation profiles. Most NF1-associated LGGs displayed a partial response. One co-occuring plexiform neurofibroma showed a volume reduction of 26% under treatment whereas other non-LGG tumors did not. As already demonstrated for selumetinib, some individuals showed tumor progression after treatment, nevertheless re-challenge seemed to be an option. In two other studies, selumetinib led to a partial response in up to 40% of recurrent LGGs and to a high percentage of progression free survivals. Only one patient had a progressive disease [42, 43]. TRAM-01 (NCT03363217) is a current prospective phase 1 study based on significant responses to trametinib in patients with refractory pediatric LGG [44]. Trametinib was also successfully applied in 5 pediatric cases with NF1 or *NF1* mutated LGGs [45–47] as well as in single small cohorts [48].

### MEKi treatment of high grade NF1 associated tumors

NF1 patients are predisposed to malignant tumors such as malignant peripheral nerve sheath tumors, glioblastomas, breast cancers, juvenile myelomonocytic leukemia, lymphoblastic leukemia, pheochromocytomas, and rhabdomyosarcomas.

Although predisposition to LGG is more common, NF1 patients are also at risk for high grade gliomas [49]. In contrast to LGG, high grade gliomas harbor a distinct molecular landscape and are enriched in *tumor protein 53* (*TP53)*, cyclin dependent kinase inhibitor 2A (*CDKN2A), alpha-thalassemia/mental retardation, X-linked (ATRX)* and t*elomerase reverse rranscriptase* (*TERT)* alterations as well as in the chromatin regulation and PI3K pathway alterations [39, 50–52]. In a mouse model, proliferation of malignant glial tumor cells has been shown to depend on MEK as well as PI3K signalling pathways and manifestation of tumors does not depend on a particular germline pathogenic NF1 variant [39, 53]. DNA methylation profiles indicate that NF1 associated gliomas belong to a poorly defined Isocitrate dehydrogenase 1 wild-type subgroup (LGm6, mesenchymal subtype) of sporadic gliomas [39]. MEKi trametinib therapy of NF1 associated high grade gliomas is reported only in single NF1 adult cases: in a 24-year-old male with NF1 and treatment-refractory glioblastoma and in a 19-year-old male with NF1 and a recurrent mesencephalic glioblastoma [54, 55]. In-vitro studies using cell lines, glioblastoma 3D oncospheres or precursor cells demonstrate sensitivity of tumor cells or of mesenchymal transition due to MEKi [52, 56–59].

NF1 associated MPNST are aggressive and infiltrative tumors characterized by high recurrence rates and early metastases. They are responsible for decreased life expectancy in NF1. They derive from benign plexiform neurofibromas and some NF1 patients are at increased risk [21]. So far, sufficient therapies do not exist, and surgical complete resection with adequate margins followed by adjuvant therapies is still the most important protective measure [60]. Loss of *CDKN2A/CDKN2B* genes in tumor Schwann cells first leads to development of premalignant, atypical neurofibromatosis neoplasms of uncertain biological potential (ANNUBP) [61]. MPNST arise when further somatic alterations occur in *Polycomb repressive complex 2 component (PRC2)* genes (that cause loss of histone H3 lysine 27 trimethylation) as well as in *TP53*. Many pre-clinical and clinical approaches to target MPNST have been followed and cannot be fully reviewed here [62]. In murine models, sarcomas generated by loss of *NF1* were sensitive to MEKi [63]. Among those, combination of MEKi with other drugs to catch multiple signaling pathways seems most promising. Experimental combination therapies include treatment of human MPNST cells with MEKi PD0235901 and all-trans retinoic acid, with MEKi and bone morphogenic protein 2 type I receptor inhibitor, with MEKi and Src homology region 2 domain-containing phosphatase-2 inhibitor and others [64–66]. Presently, only one trial recruits NF1 patients with MPNST for therapy with MEKi selumitinib in combination with mTOR inhibitor (NCT03433183). Recently it was demonstated that activation of receptor tyrosine kinases HGF/MET mediated resistance to MEKi in MPNST, and points towards a useful combination of MEK and MET inhibition NF1 patients with MPNST [5].

Although rare, NF1 associated leukemias are currently investigated in single trials using trametinib (NCT04439318, NCT03190915). Study reports have not been published so far. 

Specific knowledge of the individual genetic landscape in any high grade NF1 associated tumor by comprehensive molecular characterization will drive selection of targeted drugs beyond current approaches and will influence choice of personalized combination therapy.

### MEKi treatment of bone abnormalities in Neurofibromatosis type 1

NF1 associated bone dysplasias comprises idiopathic scoliosis, osteopenia, tibial dysplasia, short stature, pseudarthrosis, sphenoid wing dysplasia, and chest wall deformaties [67, 68]. Therapies are still challenging and patients often need repetitive surgery. Alike in other benign NF1 associated lesions, inactivation of both *NF1* gene copies is present in skeletal abnormalities due to a “second hit” leading to overactivation of the RAS-MAPK signaling cascade [69–72]. This affects early bone osteoblasts and impairs bone formation and fracture healing [73–75]. Additionally, ERK was shown to be important for osteoclast functions [76]. Deletion of *NF1* in osteoprogenitor cells in mice led to upregulation of inorganic pyrophosphate pathway related genes which finally inhibited hydroxyapatite formation and bone mineralization dependent of MEK [77]. Limited pre-clinical studies demonstrated induction of osteoblast differentiation and bone healing with combined MEKi PD98059 or trametinib and bone morphogenetic protein 2 treatment [73, 75, 77]. Recently, it was hypothesized that treatment of neurofbromas with MEKi may also improve skeletal lesions since selumitinib positively affected dysregulation of pyrophosphate homeostasis in adjacent NF tumors and partially rescued reduced tumor-associated bone mineral density in a patient [78]. In a murine fracture model of NF1 pseudarthrosis MEKi therapy with PD0325901 in combination with bone morphogenetic protein 2 led to significantly increased bone volume [79]. Recently, combination of MEKi PD0325901 with bisphosphonate zoledronic acid improved bone morphogenetic protein 2 induced spine fusion in a modified murine NF1 model [80]. In these experiments, MEKi increased bone volume and bisphosphonate zoledronic acid increased bone density versus bone morphogenetic protein 2 alone indicating a complex interaction of bone formation, deposition of fibrous tissue and repair. Beside the role of RAS-MAPK cascade in bone abnormalities in NF1 other signalling pathways such as aberrant jun N-terminate kinase (JNK) activity seem to be important for osteogenesis in NF1 [81]. These preclinical studies indicate that MEKI may be a powerful tool for therapy of severe orthopaedic problems in NF1 which would substantially improve life quality.

## Conclusions

MEKi therapy has become an important and highly selective tool to neutralize mutational events of the RAS-MAPK signaling cascade affected in Neurofibromatosis.

They seem especially promising for therapy of Neurofibromatosis type 1 patients with refractive low grade gliomas, inoperable plexiform and other neurofibromas as well as other non-tumor lesions that mainly depend on RAS-MAPK overactivation. Malignant tumors that harbor pathogenic *NF1* variants additionally to other genetic events show increased response when MEK inhibition is combined with other therapeutics.

## Data Availability

Data sharing is not applicable to this article as no datasets were generated or analyzed during the current study. Aall information in this review can be found in the reference list.

## Abbreviations

ANNUBP: Atypical neurofibromatosis neoplasms of uncertain biological potential
ATRX: Alpha-thalassemia/mental retardation, X-linked
AKT1: Serine/threonine protein kinase 1
ATP: Adenosine tri-phophate
BRAF: B-RAF
CALM: Café-au-lait macules
CDKN2A/B: Cyclin dependent kinase inhibitor 2A/B
FDA: Food and drug administration
FGFR1: Fibroblast growth factor receptor 1
ERK: Extracellular signal-related kinase
GAP: GTPase activating protein
GTP: Guanosine tri-phosphate
GRD: GAP related domain
HGF: Hepatocyte growth factor
JNK: Jun N-terminate kinase
KRAS: Kirsten rat sarcoma virus
LGG: Low grade glioma
LZTR1: Leucine zipper like transcription regulator 1
MAP: Mitogen activated protein kinase
MAPK: Mitogen activated protein kinase kinase
MAPKK: Mitogen activated protein kinase kinase (MAP 3 K)
MEK: MAP/ERK kinase
MEKi: MEK inhibitor
MET: HGF receptor
MPNST: Malignant peripheral nerve sheath tumor
NF1: Neurofibromatosis type 1
NF2: Neurofibromatosis type 2
PI3K: Phosphatidylinositol 3-kinase
NRAS: Neuroblastoma RAS
PRC2: Polycomb repressive complex 2
PTEN: Phosphatase and tensin homolog
PP2A: Protein phosphatase 2
RAF: Rapidly accelerated fibrosarcoma
RAS: Rat sarcoma
SMARCB1: SWI/SNF related, matrix associated, actin dependent regulator of chromatin, subfamily B, member 1
STAT: Signal transducer and activator of transcription
TP53: Tumor protein 53
TERT: Telomerase reverse transcriptase
